# NS1619 Alleviate Brain-Derived Extracellular Vesicle-Induced Brain Injury by Regulating BKca Channel and Nrf2/HO-1/NF-ĸB Pathway

**DOI:** 10.1155/2022/2257427

**Published:** 2022-11-23

**Authors:** Yalong Gao, Hejun Zhang, Xiaotian Li, Lei Li, Fanjian Li, Tuo Li, Ruilong Peng, Cong Wang, Jiwei Wang, Xiao Liu, Shu Zhang, Jianning Zhang

**Affiliations:** ^1^Key Laboratory of Post-Neurotrauma Neurorepair and Regeneration in Central Nervous System, Ministry of Education and Tianjin Neurological Institute, Tianjin Medical University General Hospital, 154 Anshan Road, Tianjin 300052, China; ^2^Department of Neurosurgery, Tianjin Medical University General Hospital, 154 Anshan Road, Tianjin 300052, China; ^3^Department of Neurosurgery, Tianjin Huanhu Hospital, 6 Jizhao Road, Tianjin 300060, China

## Abstract

Brain induced extracellular vesicle (BDEV) elevates after traumatic brain injury (TBI) and contributes to secondary brain injury. However, the role of BDEV in TBI remains unclear. In this study, we determined the mechanisms of BDEV in brain injury and explored whether neuroprotective drug BKca channel opener NS1619 may attenuate BDEV-induced brain injury. We injected BDEV and lactadherin, respectively, to mimic the up and downregulation of BDEV after TBI and illustrated the role of BDEV in vivo. In vitro, the membrane potential and calcium concentration of HT-22, bEnd3, and BV-2 were measured by fluorescent staining. The effects of BDEV and NS1619 on HT-22 were evaluated by CCK-8, LDH release assay, Na^+^/k^+^-ATPase activity, JC-1 staining, DHE staining, and 4-HNE staining, respectively. The role of BDEV and NS1619 on the Nrf2/HO-1/p65 pathway was also evaluated in HT-22. Finally, we administrated TBI mice with NS1619 to clarify the role of NS1619 against BDEV in vivo. Our results suggested that BDEV aggravated and lactadherin mitigated TBI-induced EB leakage, brain edema, neuronal degeneration, apoptosis, ROS level, microgliosis, MMP-9 activity, and NF-*κ*B activation. In vitro, BDEV-caused depolarized membrane potential and calcium overload were significantly attenuated by NS1619 in HT-22, bEnd3, and BV-2. BDEV markedly decreased cell viability, Na^+^/k^+^-ATPase activity, and caused mitochondrial dysregulation, oxidative stress, and NF-ĸB activation. NS1619 pretreatment alleviated above process and enhanced antioxidant system Nrf2/HO-1 in HT-22. Finally, NS1619 administration significantly inhibited neuroinflammation response and improved TBI outcome after TBI. NS1619 treatment also reduced 4-HNE content and NF-ĸB activation and enhanced Nrf2/HO-1 pathway. Our data showed that BDEV aggravated brain injury by perturbing cell membrane potential, calcium homeostasis, oxidative stress, and neuroinflammation. The BKca channel opener NS1619 attenuated BDEV-induced pathological process in vitro and in vivo by modulating the BKca channel and Nrf2/HO-1/NF-ĸB pathway.

## 1. Introduction

Traumatic brain injury (TBI) is a common cause of injury-induced death and long-term disability worldwide [1]. Current research focuses on secondary brain injury, which mainly includes neuroinflammation, oxidative stress, cortical depolarization, and neuronal calcium overload [2]. After TBI, a variety of cellular and molecular responses result in disruption of calcium homeostasis, dysregulated membrane potential, and mitochondrial dysfunction in neuronal cells [3]. These pathological changes are profound in the survival of neuron. It is meaningful that we explore the mechanisms behind the membrane potential dysfunction and calcium dysregulation after TBI.

The role of extracellular vesicle (EV) in brain injury has been emphasized. EVs are composed of a phospholipid bilayer and internal “cargos” and are considered an additional mechanism for intercellular communication. Brain derived extracellular vesicle (BDEV) released by damaged brain after TBI are recognized as the important pathobiological features of secondary brain damage [4]. Excessive production of BDEV is associated with coagulopathy, neuroinflammation, and cerebral vasospasm [5–7]. A previous study showed that lactadherin improved BDEV-induced coagulopathy and prognosis in TBI mice by promoting the clearance of BDEV [8]. Lactadherin, also known as MFG-E8, has been demonstrated to act as an intermediary between apoptotic cells in the tissue and phagocytes and plays an important role in the clearance of cell debris. Another research found that BDEV contributed to neuroinflammation and lactadherin reduced BDEV induced brain injury after stroke [9]. Although some studies suggest that BDEV is involved in secondary brain injury after TBI, the involved mechanism and the effect of BDEV on neuronal cells remain unclear.

Typically, the cellular membrane potential around the area of the injury is affected following TBI. However, it remains unclear how TBI causes neuronal depolarization [10]. Cortical depolarization enhances neuronal excitability, which may lead to excessive release of excitatory neurotransmitters and intracellular Ca^2+^ overload [11]. Cytoplasmic Ca^2+^ overload may activate protease triggering calpain-mediated proteolysis of cytoskeletal protein and causes cellular pathology [12]. Additionally, Ca^2+^ overload in neuronal cell causes mitochondrial dysfunction, excessive reactive oxygen species (ROS), and oxidative stress. Inhibition of neuronal membrane potential depolarization and Ca^2+^ overload can effectively improve the prognosis of TBI [13, 14]. Elevated Ca^2+^ in the cytoplasm mainly comes from calcium released from calcium stores and extracellular calcium influx through L-type voltage-sensitive channel. The inositol trisphosphate receptor (IP_3_R) and ryanodine receptor (RyR) are major calcium release channels in the endoplasmic reticulum and are essential in the regulation of calcium homeostasis [15]. The voltage-gated calcium channel blockers as well as the endoplasmic reticulum calcium channel blockers, 2-aminoethoxydiphenyl borate (2-APB), and thapsigargin (TG) have yielded beneficial results in basic studies [16–18]. However, an early clinical study found that nimodipine, an L-type calcium channel blocker, showed no benefit in TBI patients, adding confusion to the mechanism of calcium influx mediated neuronal damage [19]. Recent studies showed that BDEV opened calcium channel of smooth muscle cell, which has sparked our interest in exploring the role of BDEV in brain injury [7].

The large-conductance calcium- and voltage-activated K^+^ channels (BKca) are ubiquitously expressed on the plasma membrane of nervous system cell including microglia, endothelia, and smooth muscle cell, where they functionally regulate membrane potential, action potential duration, and intracellular calcium homeostasis [20, 21]. The BKca channel provides an important negative-feedback system for calcium entry into neuronal cell. NS1619, a potent activator of the BKca channel, has demonstrated significant protection through preconditioning mediated oxidative stress regulation, anti-inflammation, stabilizing membrane potential, and regulating immunity [22–25]. However, the protective effect of NS1619 on BDEV mediated brain injury remains unclear.

## 2. Materials and Methods

### 2.1. Mice and Experimental Groups

Male C57BL/6 mice (23–25 g) were purchased from the Vital River Laboratory Animal Technology Co., Ltd (Beijing, China). The experimental procedures were approved by the Experimental Animal Ethics Committee of Tianjin Medical University. TBI mice are randomly divided into following groups: (1) + PBS, (2) + BDEV (1.0 × 10^7^), (3) + Lactadherin (400 *μ*g/kg body wt iv), and (4) TBI + NS1619 (40 *μ*g/kg body wt iv), (5) Vehicle (TBI + the solvent Dimethyl sulfoxide).

### 2.2. Fluid Percussion Injury (FPI) Model

Mice were anesthetized with isoflurane. The FPI model was prepared based on previous research [6]. Briefly, a 3 mm-diameter craniectomy was performed with the dura mater intact. A needle hub was sealed over the craniectomy and linked to the FPI device. The pendulum angle of the FPI device was adjusted to achieve a peak pressure of about 1.9 ± 0.2 atmospheres onto the dura through the hub. After the experiment, the mouse was immediately removed from the apparatus, and the wound was sutured closed. Sham group received the same process except for the release of the pendulum. Immediately, after injury, the sham group and TBI group were administered saline, and the intervention group was administered BDEV, lactadherin or NS1619.

### 2.3. Collection of BDEV and Quantification by Flow Cytometry and BCA

Three hours after FPI, BDEV was obtained from freshly isolated brain lesion area according to the previous methods with appropriate modification [6, 26]. After being frozen, the tissues were sliced quickly and then incubated in DMEM medium containing 20 U/ml papain (Merck, USA) for 15 min at 37°C. Cold DMEM was then added to stop digestion. The dissociated tissue was spun at 1,500 × g for 20 min at 4°C to remove cells. Cell-free supernatant was spun at 13,000 × g for 2 min at 4°C to deplete cellular debris. The 13,000 × g supernatant was then purified by size exclusion chromatography and concentrated by ultracentrifugation at 100,000g for 2 hours at 4°C. The supernatant was discarded, and the pellet was resuspended in PBS and used for subsequent experiments.

The level of BDEV in the brain is evaluated by its concentration and total protein content, respectively, according to previous reports [27, 28]. The concentration of BDEV was determined by flow cytometry (LSR Fortessa, BD, USA). Briefly, the BDEV was identified by its size (0.1–1.0 *μ*m) using Megamix polystyrene beads (0.5, 0.9, and 3 *μ*m, Megamix-Plus SSC, Biocytex, Marseille, France) to gate the EV region by forward and side scatter. AccuCount Ultra Rainbow Fluorescent Particles (3.8 micron, Spherotech, Lake Forest, IL, USA) were used to quantify the number of BDEV. The protein content of BDEV was determined using BCA assay kit (Solarbio Science & Technology Co., Ltd, Beijing, China). Briefly, BDEV was diluted 5–10 times and was lysed with RIPA buffer. Colour was developed for 20 min at 37°C, and absorbance at 562 nm was measured. The concentration of BDEV can be obtained from the standard curve.

### 2.4. Immunofluorescence (IF) Staining

The frozen tissue sections or cultured cells were blocked by 5% bovine serum albumin with 0.1% Triton X-100, which were further incubated at 4°C overnight with the following primary antibodies: MMP9 (1: 500, Santa Cruz, USA), p65 (1: 500, Abcam, UK), HO-1(1: 500, Abclonal, China), Nrf2 (1: 500, Novus, USA), NeuN (1: 500, Abcam, UK), 4-HNE (1: 500, Abcam, UK), Iba-1 (1: 500, Abcam, UK), iNOS (1: 500, CST, USA), Arginase-1 (1: 500, CST, USA), and GFAP (1: 500, Abcam, UK). Then, cells or slides were incubated with corresponding secondary antibodies Alexa Fluor 594 and/or Alexa Fluor 488. Cells or slides were then washed again, and nuclei were counterstained with 4′,6-diamidino-2-phenylindole (DAPI, Abcam, UK) and sealed with a coverslip. Fluorescence images were taken with a fluorescence microscope (Olympus BX61, Japan) and the obtained images were analyzed with ImageJ software.

### 2.5. Western Blot (WB)

Brain tissue collected from around the injured area was homogenized in ice-cold lysis buffer supplemented with protease and phosphatase inhibitors. Total protein was quantified with the BCA assay (Solarbio, Beijing, China). The PVDF membranes were incubated with the primary antibodies against: Nrf2 (1 : 1000, Novus, USA), cleaved-caspase3 (1: 1000, CST, USA), HO-1 (1 : 1000, Abclonal, China), p-p65 (1 : 1000, Abcam, UK), and *β*-actin (1 : 3000, ZSGB, China) at 4°C overnight. Then the membranes were incubated with horseradish peroxidase-conjugated anti-mouse or anti-rabbit secondary antibody (1 : 5000, ZSGB, China) and visualized by enhanced chemiluminescence (ECL) solution (Millipore, USA). Images of blots were detected with ChemiDoc Touch Imaging System and the bands were quantified with ImageJ software.

### 2.6. Enzyme-Linked Immunosorbent Assay (ELISA)

Mice were sacrificed 3 days after TBI, and the brain tissue homogenates were obtained from the injured area. Inflammatory factors were detected using ELISA kits for tumor necrosis factor-*α* (TNF-*α*), IL-1*β*, IL-6, and IL-10 (all from R&D Systems, USA). Measured OD values were converted into a concentration value.

### 2.7. Cerebral Edema

Cerebral edema was determined by measuring brain water content with the wet-dry method 3 days after TBI. Bilateral hemispheres were separated and weighed immediately to get wet weight. After drying in the thermostat, the tissues were reweighed to get the dry weight. Brain water content (%) = (wet weight–dry weight)/wet weight × 100%.

### 2.8. Assessment of Cerebrovascular Permeability

Evans blue (EB) extravasation was used to determine blood-brain barrier (BBB) integrity. Briefly, 2% EB dye (100 *u*l/mouse, Sigma, USA) was injected via the tail vein. Two hours later, the mice were anesthetized and perfused, and the brain was rapidly harvested. The brain tissue was homogenized and centrifuged to remove the debris. The supernatants were used to detect the content of EB at 633 nm using a multifunctional microplate reader (Thermo, USA).

### 2.9. TUNEL Staining

The in situ cell apoptosis detection kit (Beyotime, China) was employed to determine the apoptosis in the brain according to the manufacturer's manual. Briefly, the slides were incubated in 0.5% Triton X-100 for 20 minutes to permeabilize tissues, followed by incubation with TUNEL reagent mixture for 1 h at 37°C. The slides were then counterstained with DAPI for 10 minutes, mounted, and viewed by the fluorescence microscope (Olympus BX61, Japan).

### 2.10. Tissue and Cellular DHE Staining

Frozen brain slices or living cells were incubated with dihydroethidium (DHE, 10uM) (Sigma, USA) fluorescent dyes at 37°C for 40 min. Then, the slices nuclei were counterstained with DAPI for 10 min. The cells were trypsinized into individual cells for flow cytometry. The images were observed using a fluorescence microscope, and the red fluorescence reflected the ROS level. The DHE-positive cells were quantified by ImageJ. The ROS content in the living cells was expressed by the mean fluorescence intensity (MFI) of DHE.

### 2.11. FJC Staining

Frozen brain slices were processed for Fluoro-Jade C (FJC) staining (Biosensis, USA) following the manufacturer's instructions. Briefly, brain slices were mounted on gelatin-coated slides and heated at 57°C for 30 mins. Slides were rehydrated with serially diluted EtOH and then blocked with KMnO_4_ solution. Then, the tissues were incubated with FJC solution containing DAPI. Tissues were dehydrated with Xylene. Images were acquired using a fluorescence microscope, and FJC-positive cells were analyzed by ImageJ software.

### 2.12. Neurologic Function Testing

The modified neurologic severity score (mNSS) test was used to determine neurologic function. The mNSS score includes motor (muscle status and abnormal movement), sensory (visual, tactile, and proprioceptive), reflex, and balance tests and is graded on a scale of 0 (normal) to 18 (maximal deficit). Neurological function was evaluated on days 1, 3, and 5 after TBI by investigators who were blinded to group information.

### 2.13. Rotarod Test

The rotarod protocol was modified minorly from that in a previous report [29]. Briefly, mice underwent a 2-day testing phase with a rotarod, which gradually accelerated from 5 to 40 rpm within 5 min. The blinded experimenter recorded the latency to fall. Average latency of 3 times in one day represented the mouse motor performance. The test was performed on 1, 3, and 5 days following TBI.

### 2.14. Cell Counting Assay

Cell Counting Kit 8 (CCK-8) assay was used to evaluate the cell viability (Beyotime, China). HT-22 was cultured on 96-well plates with 1 × 10^4^ cells per well. Twelve hours after seeding, the neurons were treated with BDEV (1^∗^10^4^/ul), NS1619 (40 uM), or vehicle for 6 hours or 12 hours. After that, 10 *μ*l CCK-8 solution was added to each well, and the cells were incubated for 1 h at 37°C. Measure the absorbance value at 450 nm using the microplate reader, and the optical density (OD) value was used to calculate cell viability by setting the control as 100%.

### 2.15. LDH Release Detection

The measurement of lactate dehydrogenase (LDH) release was conducted using a commercially available kit (Solarbio, China) according to the manufacturer's instructions. For the HT-22, supernatant from serum-free media was centrifuged to remove debris. The supernatant was then transferred to a 96-well plates, and the reaction mixture was added and incubated in the dark for 30 min at room temperature. LDH concentration was quantified by measuring the OD value at 490 nm.

### 2.16. Cell Membrane Potential and Calcium Concentration Detection

The cell membrane potential or intracellular calcium concentration was detected using the membrane potential-sensitive dye DiBAC4(3) (AAT Bioquest, USA) or Fluo-4 AM (Thermo, USA), respectively. DiBAC4 (3) itself is nonfluorescent, and only fluoresces when it binds to proteins in the cytoplasm. Intracellular fluorescence intensity increases as membrane potential increases, which indicates cellular depolarization. Cells were cultured on 96-well plates with 1 × 10^4^ cells per well. Twelve hours after seeding, the cells were stained with DiBAC4(3) (5 *u*M) or Fluo-4 AM (2 *u*M), respectively. Then, the plate was incubated at 37°C in the dark for 40 min and analyzed by a microplate reader immediately.

### 2.17. Na^+^/K^+^ ATPase Activity Assay

To investigate whether BDEV altered the Na^+^/K^+^ -ATPase activity of HT22, a Na^+^/K^+^ -ATPase activity kit was applied according to manufacturer's instructions (Solarbio, China). Briefly, the obtained HT-22 was homogenized and sonicated to release the protein. The cellular homogenates were then centrifuged, and the supernatants were collected to determine the Na^+^/K^+^-ATPase activity. The activity was calculated by subtracting the ouabain-sensitive activity from the total activity (in the absence of ouabain). Release of dinorganic phosphate (Pi) was spectrofluorimetrically measured at 650 nm, as described by a previous study [30], and Na^+^/K^+^-ATPase activity was expressed as nmol Pi/mg protein/min.

### 2.18. Detection of Cellular Phosphatidylserine (PS) Expression

The eversion of PS is one of the typical features of apoptosis. The apoptosis of cultured HT-22 was stained by FITC-labeled lactadherin (Haematologic Technologies, USA). Briefly, FITC-labeled lactadherin was added to the culture medium, incubated for 30 min at 37°C, and then washed to remove excess dyes. The nuclei were labeled with hochest 33342 (Solarbio, China). After being washed, Petri dishes were observed under an inverted fluorescence microscope.

### 2.19. MDA Content

The content of malondialdehyde (MDA) in the brain was determined by MDA kit (Solarbio, China). Three days after TBI, the brain tissues around the injured area were homogenized for the determination of MDA content.

### 2.20. Mitochondrial Membrane Potential

We measured the mitochondrial membrane potential (ΔΨm) using JC-1 fluorescence mitochondrial staining assay according to the manufacturer's instructions (Beyotime, China). Cultured HT-22 was treated with BDEV or BDEV + NS1619 (pretreatment, 40 *μ*M) for 12 hours, and then the HT-22 ΔΨm was determined by measuring changes in JC-1-derived fluorescence from red (J-aggregates) to green (monomeric) using fluorescence microscopy.

### 2.21. Statistical Analysis

All values are presented as mean ± standard deviation (SD). One-way analysis of variance with Tukey's post hoc test was used when more than two groups are being compared. Data were analyzed with GraphPad Prism 8 statistic software (La Jolla, CA). *p* < 0.05 was considered statistically significant.

## 3. Results

### 3.1. BDEV Aggravated and Lactadherin Treatment Attenuated BBB Leakage and Brain Edema after TBI

**Figure 1:**
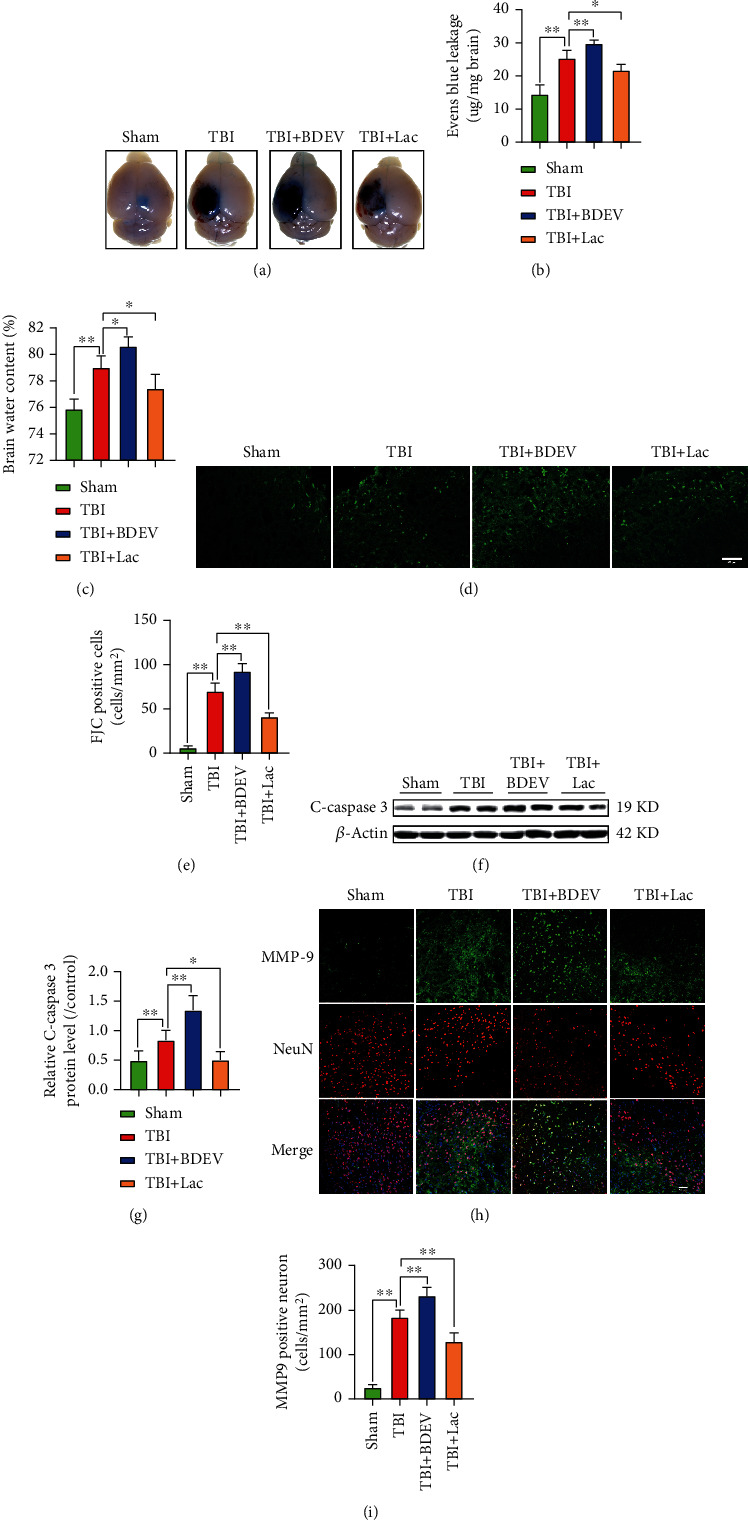
BDEV aggravated and Lac mitigated cerebral damage 3 days after TBI. (a) BBB permeability was estimated using EB leakage assay. (b) Quantitative analysis of EB content in injured regions (*n* = 6/group). (c) The content of cerebral edema in ipsilateral and contralateral regions (*n* = 6/group). (d, e) Representative images of FJC labeling (green) and quantitation of FJC positive cells (*n* = 6/group, scale bar = 50 *μ*m). (f, g) Representative WB bands and quantification of relative protein expression for cleaved caspase-3 (*n* = 7/group). (h, i) Typical double immunofluorescence images of NeuN (red) and MMP9 (green), and quantitation of double-positive cells (*n* = 6/group, scale bar = 50 *μ*m). Data are shown as mean ± SD. ^∗^*p* < 0.05, ^∗∗^*p* < 0.01.

To evaluate the effects of BDEV and lactadherin treatment on BBB integrity 3 days after TBI in mice, an EB assay was employed. The results showed that injection of BDEV significantly aggravated EB leakage, and administration of lactadherin significantly reduced EB leakage in the peri-injury area when compared to the TBI group, respectively (Figures 1(a) and 1(b)). Compared with the TBI group, injection of BDEV significantly increased ipsilateral cerebral edema content, and lactadherin treatment significantly reduced ipsilateral cerebral edema content (Figure 1(c)).

### 3.2. BDEV Aggravated and Lactadherin Treatment Attenuated Neuronal Injury and Apoptosis after TBI

To evaluate the effects of BDEV and lactadherin on neuronal injury, we evaluated by counting FJC-positive cells in the cortex. BDEV injection significantly resulted in greater neuronal injury compared to PBS-treated TBI mice. Lactadherin treatment significantly attenuated neuronal injury compared to TBI mice treated with PBS (Figures 1(d) and 1(e)). WB analyses also demonstrated that BDEV injection resulted in the upregulation of cleaved caspase-3 in the brain compared to the PBS-treated TBI group. However, lactadherin treatment significantly decreased the level of cleaved caspase-3 in the brain compared to the PBS-treated TBI group (Figures 1(f) and 1(g)).

### 3.3. BDEV Increased and Lactadherin Treatment Reduced MMP-9 Expression after TBI

Enhanced matrix metalloproteinase-9 (MMP-9) is responsible for tight junction protein degradation and neuronal injury after TBI. Injection of BDEV after TBI significantly increased the number of MMP-9 positive neurons compared to TBI alone group. Lactadherin treatment significantly decreased MMP-9 positive neurons after TBI compared with TBI alone group (Figures 1(h) and 1(i)).

### 3.4. BDEV Increased and Lactadherin Treatment Decreased ROS and Lipid Peroxidation after TBI

**Figure 2:**
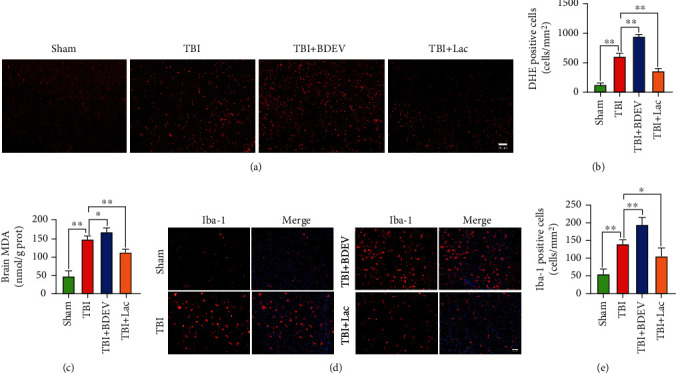
BDEV exacerbated and Lac alleviated oxidative stress and neuroinflammation 3 days after TBI. (a, b) Representative images of DHE labeling (red) and quantitation of DHE positive cells (*n* = 6/group, scale bar = 50 *μ*m). (c) MDA content in the brain (*n* = 6/group). (d, e) Representative images of Iba-1 labeling (red) and quantitation of Iba-1 positive cells (*n* = 6/group, scale bar = 50 *μ*m). Data are shown as mean ± SD. ^∗^*p* < 0.05, ^∗∗^*p* < 0.01.

We employed DHE staining and MDA content kit to quantify ROS level and lipid peroxidation content in the cortex. The results showed that injection of BDEV significantly increased DHE positive cells and lactadherin treatment significantly reduced DHE positive cells compared with PBS treated TBI group (Figures 2(a) and 2(b)). Injection of BDEV significantly increased MDA content in the brain, and lactadherin treatment significantly reduced MDA content in the brain compared to TBI mice treated with PBS (Figure 2(c)).

### 3.5. BDEV Increased and Lactadherin Treatment Reduced Inflammatory Cells after TBI

To investigate the effects of BDEV and lactadherin on the activation of inflammatory cells, we identified Iba-1 positive microglia/macrophages in the injured cortex by IF. Injection of BDEV significantly increased Iba-1 positive cells compared to TBI alone group. Lactadherin treatment significantly decreased Iba-1 positive cells compared to TBI alone group (Figures 2(d) and 2(e)).

### 3.6. BDEV Promoted and Lactadherin Treatment Inhibited the Expression of NF-*κ*B after TBI

**Figure 3:**
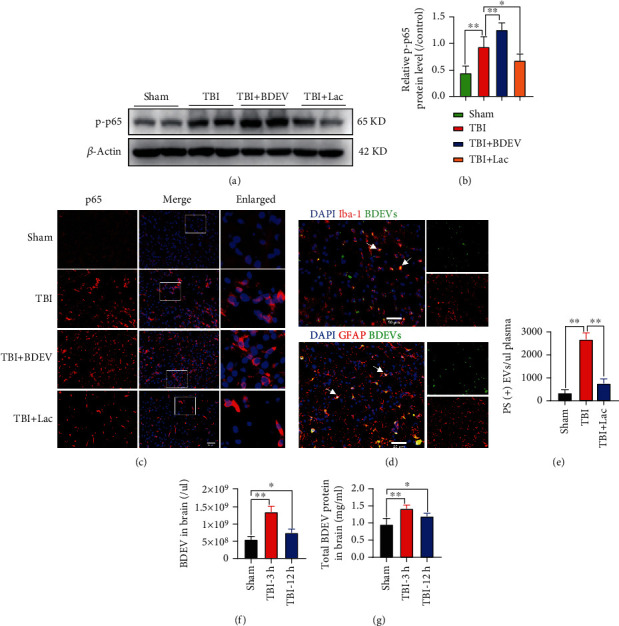
BDEV upregulated and Lac downregulated NF-*κ*B/p65 pathway 3 days after TBI. Lac mediated BDEV uptake by astrocytes and microglia. (a, b) Representative WB bands and quantification of relative protein expression for NF-*κ*B/p-p65 (*n* = 6/group). (c) Representative images of NF-*κ*B/p65 staining (red) (*n* = 3/group, scale bar = 50 *μ*m). (d) Representative images of colocalization of microglia/macrophages (red) and astrocytes (red) with BDEV (Green, FITC labeled Lac) (*n* = 3/group, scale bar = 50 *μ*m). (e) Lac reduced PS-positive EV in circulation. (f. g) The concentration and total protein content of BDEV in brain at 3 h and 12 h after TBI. Data are shown as mean ± SD. ^∗^*p* < 0.05, ^∗∗^*p* < 0.01.

To further examine the effects of BDEV and lactadherin on the inflammatory signaling pathway, the expression of p-p65 in the brain was determined by WB, and IF. BDEV significantly increased the expression of p-p65 protein in the brain compared with the TBI group. In contrast, treatment with lactadherin significantly decreased the TBI-induced upregulation of p-p65 protein. IF showed that BDEV injection significantly increased nuclear and cytoplasmic expression of p65 protein compared to the TBI group. Lactadherin treatment significantly decreased nuclear and cytoplasmic p65 protein compared to the TBI group (Figures 3(a)–3(c)).

### 3.7. Lactadherin Treatment Promoted BDEV Phagocytosis and the Level of BDEV in Brain Increased after TBI

A previous study showed that lactadherin mediated clearance of EV by the phagocytosis system. We further explored the clearance role of lactadherin in the brain. The prepared BDEV was coincubated with FITC-labeled lactadherin to fully bind with each other. Then, 1.5 × 10^7^ fluorescent BDEV was infused into a mouse through the tail vein, and the brains were dissected 12 hours after injection and processed for immunofluorescence staining to detect the endocytosis of BDEV. The results showed that BDEV mainly colocalizes with Iba-1 or GFAP-positive cells (Figure 3(c)). To reconfirm the effect of lactadherin on circulating EV, we injected TBI mice with lactadherin and detected circulating PS-positive EV 12 h after TBI. The results of flow cytometry showed that lactadherin treatment significantly reduced circulating PS-positive EV compared to PBS treated TBI group (Figure 3(e)). The level of EV is often evaluated by its concentration and total protein content. The results showed that the concentration measured by flow cytometry (Supplementary Figure 1) and total protein content of BDEV in brain markedly increased at 3 h and 12 h after TBI (Figures 3(f) and 3(g)). BDEV is abundantly produced in the acute phase after TBI.

### 3.8. BDEV-Induced Depolarized HT-22 Was Rescued by NS1619

**Figure 4:**
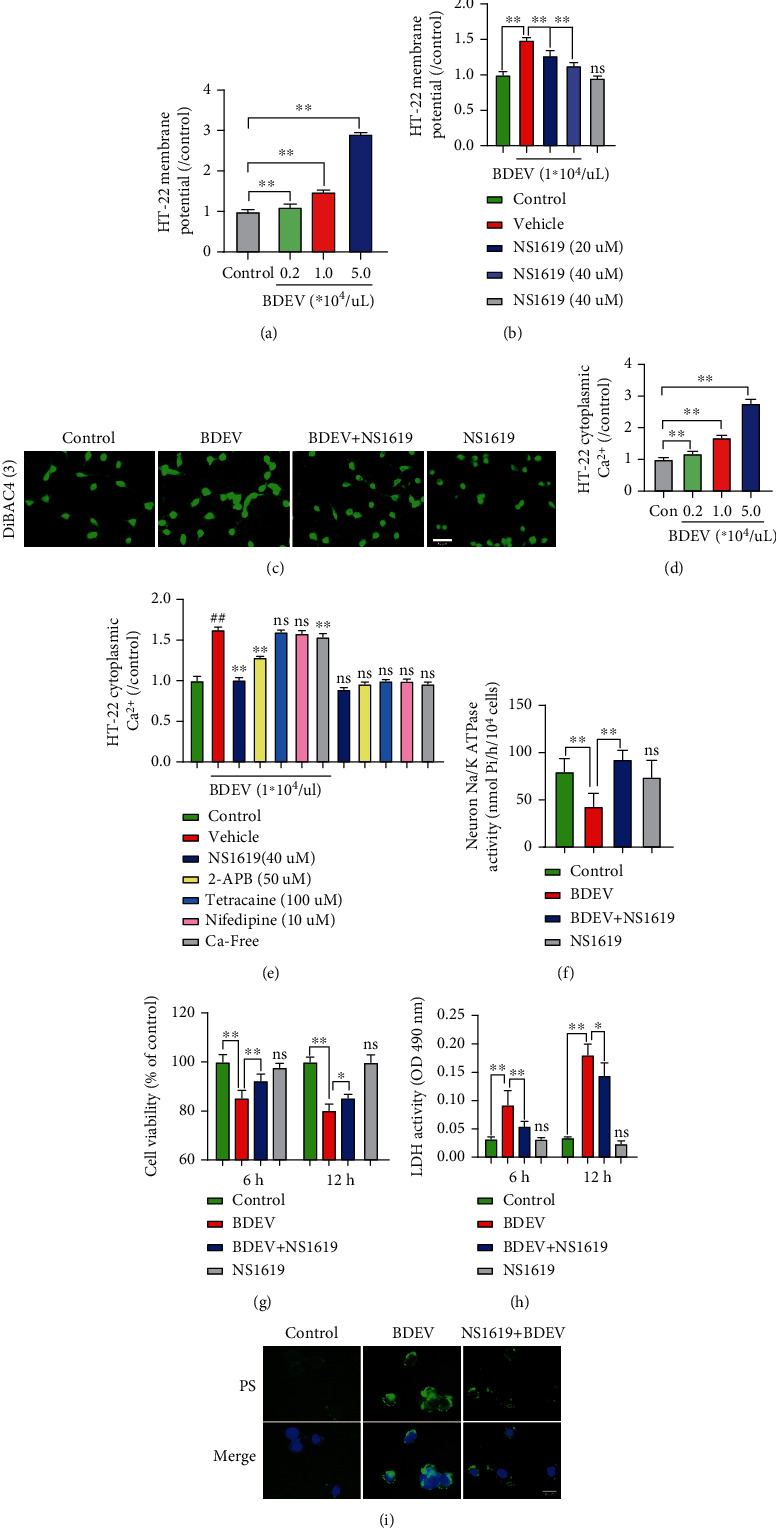
BDEV-induced cell damage was attenuated by NS1619 in HT-22. (a) BDEV induced a concentration-dependent increase of HT-22 membrane potential (*n* = 6/group). (b) NS1619 inhibited increased membrane potential induced by BDEV in HT-22 (*n* = 6/group). (c) Representative fluorescence images of DiBAC4(3) staining in HT-22 (Green, *n* = 3/group, scale bar = 50 *μ*m). (d) BDEV induced a concentration-dependent increase of calcium concentration in HT-22 (*n* = 6/group). (e) BDEV-induced cytoplasmic calcium overload was completely blocked by NS1619 and partially blocked by 2-APB and calcium-free solution, but not blocked by tetracaine and nifedipine (*n* = 6/group). (f) NS1619 restored inactivated Na^+^/K^+^-ATPase damaged by BDEV in HT-22 (*n* = 6/group). (g) NS1619 effectively ameliorated the decrease of HT-22 cell viability caused by BDEV detected by a CCK-8 assay (*n* = 6/group). (h) NS1619 effectively reduced the release of LDH in HT-22 caused by BDEV (*n* = 6/group). (i) NS1619 effectively inhibited PS eversion induced by BDEV (*n* = 3/group, scale bar = 20 *μ*m). Data are shown as mean ± SD. ns, *p* > 0.05, ^∗^*p* < 0.05, ^∗∗^*p* < 0.01.

The cultured cells were exposed to various concentrations of BDEV (0.2, 1.0, and 5.0^∗^10^4^/uL) for 3 h. BDEV significantly depolarized the HT22 membrane potential in a concentration-dependent way compared to the control group (Figure 4(a)). Both concentrations of NS1619 significantly rescued the depolarized HT-22 membrane potential induced by BDEV (Figure 4(b)). Representative fluorescence images of DiBAC4 (3) staining in HT-22 (Figure 4(c)).

### 3.9. BDEV-Induced HT-22 Ca^2+^ Overload Was Reduced by NS1619

BDEV significantly increased the HT-22 cytoplasmic Ca^2+^ fluorescence intensity in a concentration-dependent way (0.2, 1.0, and 5.0^∗^10^4^/uL) at 3 h compared to control group (Figure 4(d)). BDEV-induced cytoplasmic Ca^2+^ overload in HT-22 was completely blocked by NS1619 (40 *u*M) and partially blocked by IP3 receptor inhibitor 2-APB (50 *u*M) and calcium-free solution, but not blocked by tetracaine (100 *u*M) and nifedipine (10 *u*M) (Figure 4(e)).

### 3.10. BDEV-Induced Decreased Na^+^/K^+^-ATPase Activity Was Attenuated by NS1619 in HT-22

Since Na^+^/K^+^-ATPase controls intracellular ion homeostasis and maintains the resting membrane potential, and they are especially sensitive to calcium overload and oxidative stress, we evaluated the effect of BDEV and NS1619 on Na^+^/K^+^-ATPase activity at 12 h after treatment. Statistical analyses revealed a significant decrease in Na^+^/K^+^-ATPase activity in BDEV treated group compared to the control group, and NS1619 pretreatment significantly enhanced Na^+^/K^+^-ATPase activity compared to the BDEV group (Figure 4(f)).

### 3.11. BDEV-Induced Decreased Cell Viability Was Attenuated by NS1619 in HT-22

We employed the CCK-8 assay and LDH release assay to determine the effects of BDEV and NS1619 on neuronal viability. The CCK-8 assay showed that BDEV (1.0^∗^10^4^/*u*L) significantly decreased the neuronal viability compared to the control group when coincubated with HT-22 for 6 h and 12 h, while pretreatment with NS1619 (40 *u*M) significantly improved neuronal viability induced by BDEV (Figure 4(g)). LDH release assay showed that BDEV (1.0^∗^10^4^/*u*L) significantly increased the LDH release at 6 h and 12 h compared to the control group, and pretreatment with NS1619 (40 *u*M) significantly suppressed the LDH release compared with BDEV group (Figure 4(h)). NS1619 alone (40 *μ*M) had no significant effect on cell viability shown by the CCK-8 assay and the LDH release assay without BDEV. PS can be used as an early indicator of apoptosis when PS eversion occurs. We employed FITC-labeled Lac to determine the effects of NS1619 (40 *μ*M) and BDEV (1.0^∗^10^4^/uL) intervention on PS in HT22. Fluorescence images revealed that NS1619 pretreatment significantly reduced BDEV-induced PS eversion (Figure 4(i)).

### 3.12. BDEV-Induced Mitochondrial Dysfunction and ROS Were Attenuated by NS1619 in HT-22

**Figure 5:**
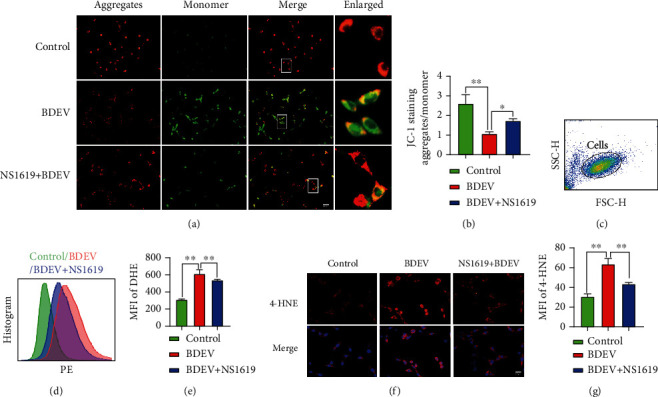
NS1619 reduced BDEV-induced *ΔΨ*m disruption, ROS release, oxidative stress products. (a, b) NS1619 pretreatment markedly improved mitochondrial membrane potential destroyed by BDEV in cultured HT-22 (*n* = 6/group, scale bar = 50 *μ*m). (c–e) Intracellular ROS level in HT-22 stained by DHE was determined by flow cytometry. NS1619 pretreatment markedly reduced ROS level caused by BDEV in cultured HT-22 (*n* = 6/group). (f, g) Representative images of 4-HNE staining (red) and statistical analysis (*n* = 3/group, scale bar = 20 *μ*m). Data are shown as mean ± SD. ^∗∗^*p* < 0.01.

The loss of mitochondrial membrane potential (*ΔΨ*m) means mitochondrial disfunction, consequently releasing ROS from mitochondria into the cytosol. HT-22 exposed to BDEV resulted in significant dissipation of *ΔΨ*m thus showing increased green fluorescence compared to the control group, which indicates the existence of monomeric JC-1 and depolarization of the mitochondrial membrane. NS1619 pretreatment significantly inhibited the dissipation of *ΔΨ*m induced by BDEV compared to the BDEV group (Figures 5(a) and 5(b)). Next, we investigated the cellular ROS level stained by DHE detected by flow cytometry. BDEV significantly increased the MFI of DHE staining in HT-22 compared to the control group. NS1619 pretreatment significantly reduced the MFI induced by BDEV in HT-22 compared to the BDEV group (Figures 5(c)–5(e)).

### 3.13. BDEV-Induced Oxidative Stress Was Attenuated by NS1619 in HT-22

We employed 4-HNE immunofluorescence staining to determine the effects of BDEV and NS1619 on oxidative stress products. BDEV significantly increased the MFI of 4-HNE in HT-22 compared to the control group. NS1619 pretreatment significantly reduced the MFI induced by BDEV in HT-22 compared with the BDEV group (Figure 5(f)).

### 3.14. BDEV-Induced Oxidative Stress Is Alleviated by NS1619 by Enhancing Antioxidant Pathways in HT-22

**Figure 6:**
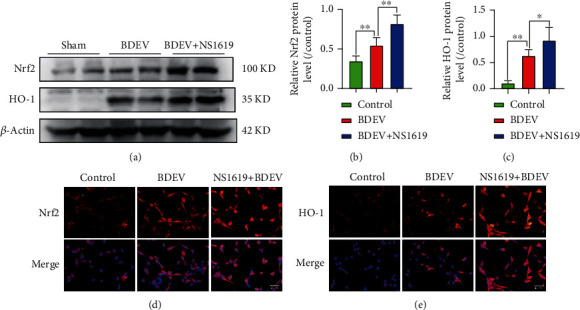
Effect of BDEV and NS1619 on the protein expression of Nrf2 and HO-1 in HT-22. Cultured HT-22 was treated with BDEV or BDEV+NS1619 (pretreatment, 40 *μ*M for 12 hr). (a–c) Representative WB bands and quantification of relative protein expression for Nrf2 and HO-1 (*n* = 6/group). (d) Representative images of Nrf2 staining (red) (*n* = 3/group, scale bar = 40 *μ*m). (e) Representative images of HO-1 staining (red) (*n* = 3/group, scale bar = 40 *μ*m). Data are shown as mean ± SD. ^∗^*p* < 0.05, ^∗∗^*p* < 0.01.

Nuclear factor E2-related factor 2 (Nrf2) is an intracellular transcriptional regulator, and heme oxygenase 1 (HO-1) is one of its most important downstream regulatory products. The cascade reaction of the two is crucial for anti-inflammatory and antioxidant systems. WB analysis showed that NS1619 pretreatment significantly increased the expression of Nrf2 and HO-1 proteins in HT-22 compared to the BDEV group (Figures 6(a)–6(c)). Moreover, immunofluorescence analysis also confirmed that NS1619 pretreatment significantly increased the translocation of Nrf2 proteins to the nucleus and cytoplasmic HO-1 in HT-22 compared to the BDEV group (Figures 6(d) and 6(e)).

### 3.15. BDEV-Induced NF-*κ*B Activation Was Inhibited by NS1619 in HT-22

**Figure 7:**
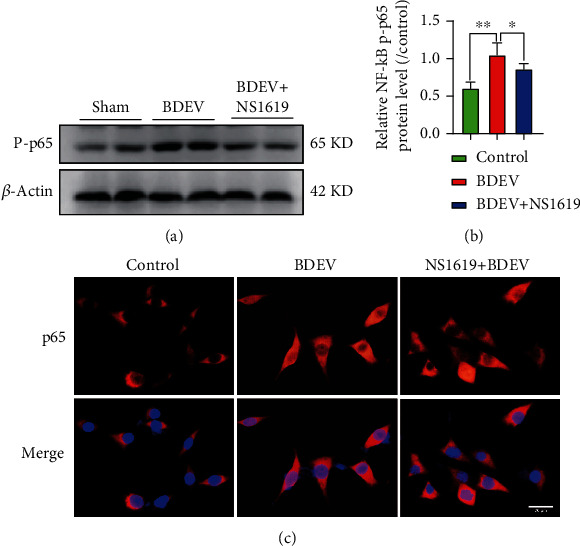
BDEV-induced NF-*κ*B/p65 activation was inhibited by NS1619 in HT-22. (a, b) Representative WB bands and quantification of relative protein expression for NF-*κ*B/p-p65 (*n* = 6/group). (c) Representative images of NF-*κ*B/p65 staining (red) (*n* = 3/group, scale bar = 20 *μ*m). Data are shown as mean ± SD. ^∗^*p* < 0.05, ^∗∗^*p* < 0.01.

NF-*κ*B/p65 pathway mediated pathophysiological process closely related to intracellular calcium overload. WB analysis showed that BDEV significantly increased the expression of p-p65 protein compared to the control group. NS1619 pretreatment significantly reduced the expression of p-p65 protein induced by BDEV compared with the BDEV group (Figures 7(a) and 7(b)). Immunofluorescence analysis also confirmed that NS1619 pretreatment significantly decreased the cytoplasmic and nuclear p65 compared to the BDEV group (Figure 7(c)).

### 3.16. BDEV-Induced Depolarization and Ca^2+^ Overload Was Rescued by NS1619 in bEnd3 and BV-2

**Figure 8:**
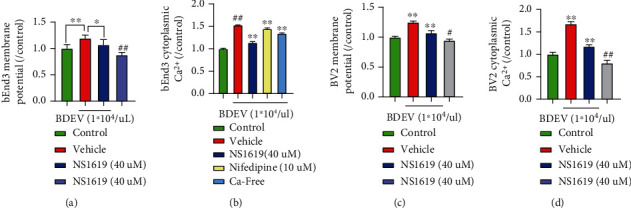
BDEV-induced membrane potential disturbance and calcium overload were attenuated by NS1619 in bEnd3 and BV-2. (a) NS1619 attenuated membrane potential depolarization induced by BDEV in bEnd3 (*n* = 6/group). (b) NS1619, nifedipine and Ca^2+^-free solution significantly ameliorated cytoplasmic calcium overload induced by BDEV in bEnd3 (*n* = 6/group). (c, d) NS1619 inhibited increased membrane potential and cytoplasmic calcium overload induced by BDEV in BV-2 (*n* = 6/group). Data are shown as mean ± SD. ^∗^*p* < 0.05, ^∗∗^*p* < 0.01. ^#^*p* < 0.05 versus the control group, ^##^*p* < 0.01 versus the control group.

BDEV (1.0^∗^10^4^/*u*L) significantly depolarized the bEnd3 membrane potential compared to the control group and NS1619 significantly rescued the depolarized bEnd3 membrane potential induced by BDEV (Figure 8(a)). BDEV significantly increased the bEnd3 cytoplasmic Ca^2+^ concentration compared to the control group. BDEV-induced cytoplasmic Ca^2+^ overload in the bEnd3 was significantly reduced by NS1619 (40 *u*M) and nifedipine (10 *u*M) and calcium-free solution (Figure 8(b)). BDEV-induced depolarization and Ca^2+^ overload was rescued partially by NS1619 in BV-2 (Figures 8(c) and 8(d)).

### 3.17. NS1619 Treatment Attenuated Neurological Impairment and BBB Damage 3 Days after TBI

**Figure 9:**
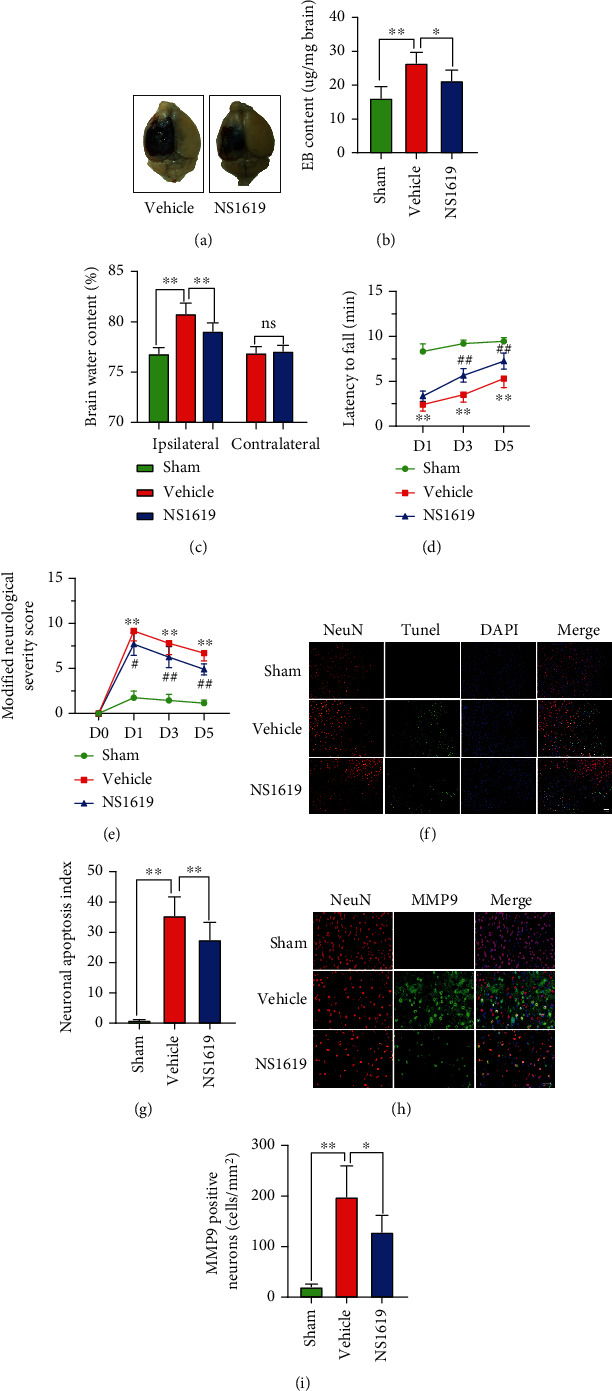
NS1619 treatment reduced EB leakage and brain edema and improved neurologic function after TBI. (a, b) NS1619 reduced EB leakage content. (c) NS1619 reduced brain edema content. (d, e) NS1619 improved motor function and neurologic deficits in TBI mice. (f, g) Representative immunofluorescence images and quantification of TUNEL (green) and NeuN (red) positive cells (*n* = 6/group, scale bar = 50 *μ*m). (h, i) Representative double immunofluorescence images of NeuN (red) and MMP9 (green), and quantitation of double-positive cells (*n* = 6/group, scale bar = 40 *μ*m). Data are shown as mean ± SD. ns, *p* > 0.05, ^∗^p < 0.05, ^∗∗^p < 0.01; ^#^*p* < 0.05, ^##^*p* < 0.01 versus the control group.

NS1619 treatment significantly decreased EB leakage and ipsilateral brain water content compared to TBI mice treated with vehicle (Figures 9(a)–9(c)). Administration of NS1619 significantly improved neurological and motor function when compared to the vehicle group (Figures 9(d) and 9(e)).

### 3.18. NS1619 Treatment Attenuated Neuronal Damage 3 Days after TBI

TUNEL staining showed that there were more apoptotic neurons in the vehicle group than in the NS1619 treated group (Figures 9(f) and 9(g)). TBI significantly increased MMP-9 positive neurons compared to the sham group, and NS1619 treatment significantly inhibited the increase of MMP-9 positive neurons compared to the vehicle group (Figures 9(h) and 9(i)).

### 3.19. NS1619 Treatment Improved Neuroinflammation 3 Days after TBI

**Figure 10:**
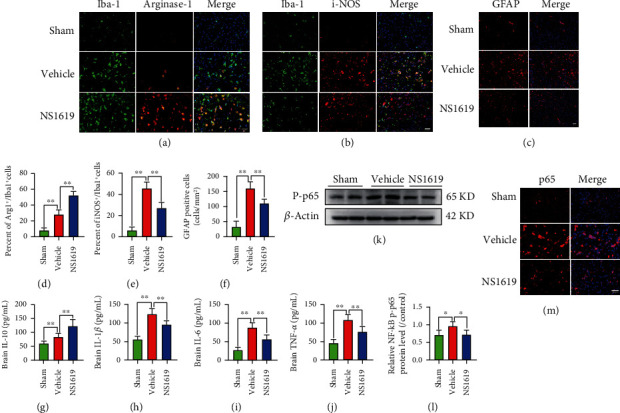
NS1619 attenuated the inflammatory response and inflammatory pathway in the brain 3 days after TBI. (a–c) NS1619 treatment markedly increased arginase1-positive microglia/macrophages and decreased iNOS-positive microglia/macrophages and GFAP-positive cells (scale bar = 20 *μ*m). (d–f) Quantification of immunofluorescence images (*n* = 6/group). (g–j) NS1619 significantly increased anti-inflammatory cytokine IL-10 but decreased proinflammatory cytokines IL-1*β*, IL-6, and TNF-*α* in brain tissue after TBI (*n* = 7/group). (k,l) Representative WB bands and quantification of relative protein expression for NF-*κ*B/p-p65 (*n* = 6/group). (m) Representative immunofluorescence images of NF-*κ*B/p-p65 staining (red) (*n* = 3/group, scale bar = 50 *μ*m). Data are shown as mean ± SD. ^∗^*p* < 0.05, ^∗∗^*p* < 0.01.

Microglia and astrocytes play an important role in neuroinflammation and secondary injury after TBI, and the effect of BDEV on glial activation after TBI has also been studied previously. We found that NS1619 treatment significantly promoted microglial/macrophage phenotypic transformation from proinflammatory M1-phenotype to anti-inflammatory M2-phenotype and significantly reduced GFAP positive cells in the peri-injury area compared to the vehicle group (Figures 10(a)–10(f)). Compared with the vehicle group, NS1619 treatment significantly increased brain IL-10 and significantly decreased brain IL-1*β*, IL-6, and TNF-*α*. (Figures 10(g)–10(j)).

### 3.20. NS1619 Treatment Inhibited NF-*κ*B Pathway Activation 3 Days after TBI

WB analysis showed that NS1619 treatment significantly reduced the expression of NF-*κ*B/p-p65 protein in the brain compared to the vehicle group. (Figures 10(k) and 10(l)). Moreover, immunofluorescence also showed that NS1619 treatment significantly reduced p65 protein localized in the nucleus and cytoplasm (Figure 10(m)).

### 3.21. NS1619 Treatment Reduced Oxidative Stress and Enhanced Antioxidant Pathway 3 Days after TBI

**Figure 11:**
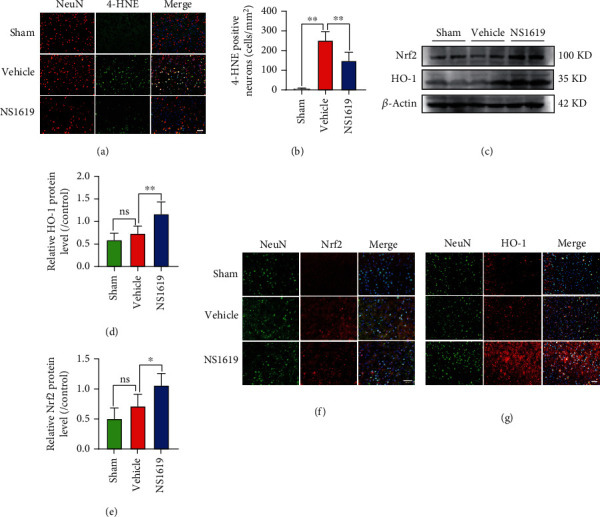
NS1619 attenuated oxidative stress and regulated antioxidant pathway 3 days after TBI. (a, b) Representative immunofluorescence images and quantification of 4-HNE (green) and NeuN (red) double-positive cells (*n* = 6/group, scale bar = 20 *μ*m). (c–e) Representative WB bands and quantification of Nrf2 and HO-1 proteins (*n* = 6/group). (f, g) Representative immunofluorescence images of Nrf2 (red), HO-1 (red) and NeuN (green) (*n* = 3/group, scale bar = 50 *μ*m). Data are shown as mean ± SD. ns, *p* > 0.05, ^∗^*p* < 0.05, ^∗∗^*p* < 0.01.

Immunofluorescence showed that NS1619 treatment significantly reduced the number of 4-HNE-positive neurons compared to the vehicle group (Figures 11(a) and 11(b)). Moreover, WB analysis showed that the relative expression level of Nrf2 and HO-1 was significantly upregulated by NS1619 after TBI compared to the vehicle group. However, TBI only slightly elevated Nrf2 and HO-1 protein compared to the control group (Figures 11(c)–11(e)). Immunofluorescence also showed that NS1619 treatment significantly increased Nrf2 translocation to the nucleus and cytoplasmic HO-1protein (Figures 11(f) and 11(g)).

## 4. Discussion

In this study, we demonstrated that BDEV injection aggravated and lactadherin treatment attenuated BBB leakage, brain edema, neuronal injury, oxidative stress, and neuroinflammation after TBI in mice. We confirmed the phenomenon earlier that BDEV caused cellular membrane potential disturbance, calcium overload, and elevated ROS and activated NF-ĸB/p65 signaling pathway in vitro. We found that BKca channel opener NS1619 significantly rescued depolarized membrane potential and calcium overload, reduced free radicals, and enhanced antioxidant pathway in vitro. Moreover, NS1619 treatment also significantly improved the prognosis of TBI mice. Our data suggested that BDEV mediated cellular ion homeostasis, and excessive ROS may contribute to brain injury after TBI.

BDEV significantly increased in biological fluids after TBI and may play an important role in the pathological development and prognosis after TBI [31, 32]. BDEV can aggravate BBB damage, neuroinflammation, and cerebral vasospasm [7, 8, 33]. Firstly, we prepared BDEV from the enzymatically dissociated brains 3 h after TBI because a previous study found that circulating BDEV peaked at this time [6]. Our data also verified that free BDEV was significantly elevated in the brain space 3 hours after TBI. To characterize the role of BDEV in brain injury, we mimicked the upregulation and downregulation of BDEV by injecting BDEV and lactadherin, respectively, which mediated the clearance of BDEV. BDEV injection significantly increased ROS and lipid peroxidation products in the brain. Expectedly, lactadherin treatment suppressed these changes. Previous studies also reported that EVs exacerbated oxidative stress. However, some studies concluded to the contrary, and they found that some cell-derived EVs protect hippocampal neurons from oxidative stress especially from stem cells [34, 35]. This suggests that BDEV released from different pathological states may be different.

TBI is known to trigger complex systemic and focal inflammatory responses [36]. The underlying mechanisms of posttrauma neuroinflammation are largely uncertain. After brain injury, locally generated BDEV disseminates into the circulation, which appears to link these inflammatory responses [9, 33]. The proinflammatory effect of BDEV on microglia has been mentioned in previous studies, so we did not further verify this result in microglia [5]. In vivo experiments, NS1619 treatment facilitated microglial/macrophage phenotypic transformation from proinflammatory M1-phenotype to anti-inflammatory M2-phenotype after TBI. This result confirmed the regulatory effect of NS1619 on inflammatory cells. Indeed, a large fraction of the microglia expressed BKca channels sensitive to the modulator NS1619 [37]. Contemporarily, NS1619 treatment increased anti-inflammatory factors and decreased proinflammatory factors in TBI mice.

Proteolytic enzymes MMP-9 are sharply elevated in the early posttraumatic period and are key mediators of trauma-associated brain edema [38]. BDEV injection significantly increased the expression of MMP-9 in neurons, and lactadherin treatment mitigated these changes. This result indicates that BDEV may exacerbate brain damage by upregulating MMP-9. Elevated MMP-9 activity caused early brain injury after TBI including BBB disruption and inflammation [39]. A key factor activated by TBI is nuclear factor-*κ*B (NF-*κ*B). Because this pathway is implicated in the mechanism of brain edema and neuroinflammation [29, 40], we examined whether NF-*κ*B might also be involved in the BDEV-related brain injury. Here, we show an increase in NF-*κ*B/p65 protein activation in the brain after injecting BDEV, and lactadherin treatment significantly decreased the trauma-induced NF-*κ*B activation.

Altered neuronal calcium homeostasis and mitochondrial dysfunction play a central role in the pathogenesis of TBI, and the mechanism behind them is still obscure. Massive neuronal depolarization occurs after TBI, which may open voltage-gated calcium channels leading to further calcium influx and cytosolic calcium overload [41, 42]. Disrupted calcium homeostasis leads to mitochondrial dysfunction and oxidative stress, an important mechanism of secondary injury after TBI [43, 44]. Previous studies focused on glutamate-mediated neuronal excitotoxicity and depolarization. However, glutamate receptor blockers have not shown satisfactory results in clinical practice [45]. This suggests that the mechanism of neuronal calcium overload induced by TBI is complex. Store-operated Ca^2+^ entry (SOCE) mediated by calcium release-activated calcium (CRAC) channels also contributes to increased intracellular calcium and CRAC channel inhibitor improves TBI outcome by inhibiting neuroinflammation [46]. Another study found that blocking IP3 receptor with 2-APB significantly reduced infarcts in a cerebral ischemia model [47]. In this study, BDEV can significantly depolarize neurons and endothelial cells and cause cytoplasmic calcium overload in vitro. BKca channel activator NS1619 effectively rescued membrane potential and calcium disturbance. However, L-type voltage-gated Ca^2+^ channel antagonist nifedipine showed few effects on calcium concentration induced by BDEV, and extracellular calcium-free solution did not completely prevent elevated calcium concentration in HT-22. These results suggest that BDEV-induced calcium overload involves the endoplasmic reticulum and is not related to L-type calcium channel. And further data also confirmed that the IP3 receptor blocker 2-APB effectively reduced cytosolic calcium concentration. Endothelial cell differs from a neuron in that nifedipine slightly blocked BDEV-induced calcium overload. From these results, we conclude that BDEV induces extracellular calcium influx and stores calcium release, which together are involved in calcium overload. Calcium overload can trigger a series of downstream prodeath signaling events, such as calpain activation, ROS generation, mitochondrial damage, etc., resulting in cell apoptosis.

BKca channel not only regulates cell membrane potential but also affects calcium concentration [48]. The activation of BKca channels limits the Ca^2+^ influx from voltage-gated Ca^2+^ channel [49]. BKca channel opener can mitigate neuronal depolarization; the elevation of intracellular Ca^2+^ and neurotransmitter release after stroke and activation of BKca channel exerts potent neuroprotection [50, 51]. Additionally, opening the mitochondrial BKca channel increased the mitochondrial membrane potential and attenuated the oxygen-glucose deprivation and reperfusion-induced upregulated cleaved caspase3 and neuronal apoptosis [52]. A previous study confirmed that BKca channel opener reduced the production of ROS in isolated rat brain mitochondria. This is consistent with our finding that NS1619 reduced mitochondrial ROS caused by BDEV [53].

Na^+^/K^+^-ATPase is one of the vital enzymes that manipulate intracellular ion homeostasis and keep the resting membrane potential and excitable properties of neurons [30]. Oxidative stress following TBI results in prolonged impairment of Na^+^/K^+^-ATPase activity, which aggravates secondary brain injury [54, 55]. In cell experiments, BDEV significantly inhibited the Na^+^/K^+^-ATPase activity. Na^+^/K^+^-ATPase inactivation may be related to changes in membrane potential.

Oxidative stress emerges when there is an inability or damage to balance the antioxidant system with excessive ROS. Oxidative stress drives many pathophysiologic changes that occur following TBI. ROS exacerbates lipid peroxidation of polyunsaturated fatty acids in cell membranes leading to the accumulation of aldehydes such as MDA, 4-hydroxy-2-nonenal (4-HNE), and other toxic substances [56]. On one hand, oxidative stress damage tight junction associated with proteins and indirectly activates MMPs that contribute to disrupting the BBB [57]. On the other hand, oxidative stress activates inflammatory cells and increases the release of inflammatory factors [58]. We found that NS1619 treatment not only reduced BDEV-induced oxidative stress products but also increased the activity of antioxidant pathways by activating Nrf2/HO-1 system. Indeed, the antioxidant function of NS1619 has been reported in an intestinal damage model [24].

Nrf2 acts as an important protective factor and a downstream target of therapeutic agents in TBI [59]. Several researches have confirmed that the nuclear shift of Nrf2 strengthens antioxidative stress, antiapoptosis, and anti-inflammation effect in TBI via different molecules and pathways including HO-1 and NF-*κ*B [60]. Although the relationship between Nrf2 and NF-*κ*B has not been fully elucidated, the putative crosstalk between the two has been found in the inflammation model [61]. In our study, BDEV injection significantly increased the nuclear translocation of p65 in vivo as well as in neuron. Unsurprisingly, the activation of NF-*κ*B was significantly reduced by NS1619 treatment in vitro and in vivo. Given the intimate relationship between BDEV and brain injury as well as its various pathogenic mechanisms, it may be a promising choice to block the BDEV's site of action in future.

## 5. Conclusion

BDEV generated after TBI exacerbates secondary brain injury by causing membrane potential disturbance, calcium overload, Na^+^/K^+^-ATPase inactivation, mitochondrial dysfunction, oxidative stress, and neuroinflammation. BKca channel opener NS1619 exerts neuroprotective effects by stabilizing cell membrane potential, reducing calcium overload, and regulating antioxidant and anti-inflammatory pathways Nrf2/HO-1/p65.

## Data Availability

The original contributions presented in the study are included in the article. Further inquiries can be directed to the corresponding author.

## Abbreviations

TBI: Traumatic brain injury
BDEV: Brain induced extracellular vesicle
ROS: Reactive oxygen species
MMP9: Matrix metalloproteinase9
4-HNE: 4-hydroxynonenal
BKca channel: Large-conductance calcium- and voltage-activated K^+^ channel
IP_3_R: Inositol trisphosphate receptor
RyR: Ryanodine receptor
2-APB: 2-aminoethoxydiphenyl borate
TG: Thapsigargin
FPI: Fluid percussion injury
IF: Immunofluorescence
GFAP: Glial fibrillary acid protein
iNOS: Inducible nitric oxide synthase
DAPI: 4′,6-diamidino-2-phenylindole
TNF-*α*: Tumor necrosis factor-*α*
BBB: Blood-brain barrier
TUNEL: TdT-mediated dUTP nick-end labeling
DHE: Dihydroethidium
MFI: Mean fluorescence intensity
PS: Phosphatidylserine
MDA: Malondialdehyde
PBS: Phosphate buffered saline
EB: Evans blue.

## Supplementary Materials

Supplementary Materials Supplementary Figure 1: the level of BDEV in brain markedly increased after TBI. The concentration of BDEV in brain is measured by flow cytometry in three groups (From left to right in the panel means, respectively: EV gate setting, sham group, 3 hours, and 12 hours after TBI).
