# Supplementary figures and images for: NS1619 Alleviate Brain-Derived Extracellular Vesicle-Induced Brain Injury by Regulating BKca Channel and Nrf2/HO-1/NF-ĸB Pathway
Source: Oxid Med Cell Longev. 2022 Nov 23;2022:2257427. doi: 10.1155/2022/2257427 (PMC9711983; doi:10.1155/2022/2257427)

Supplementary figure 1


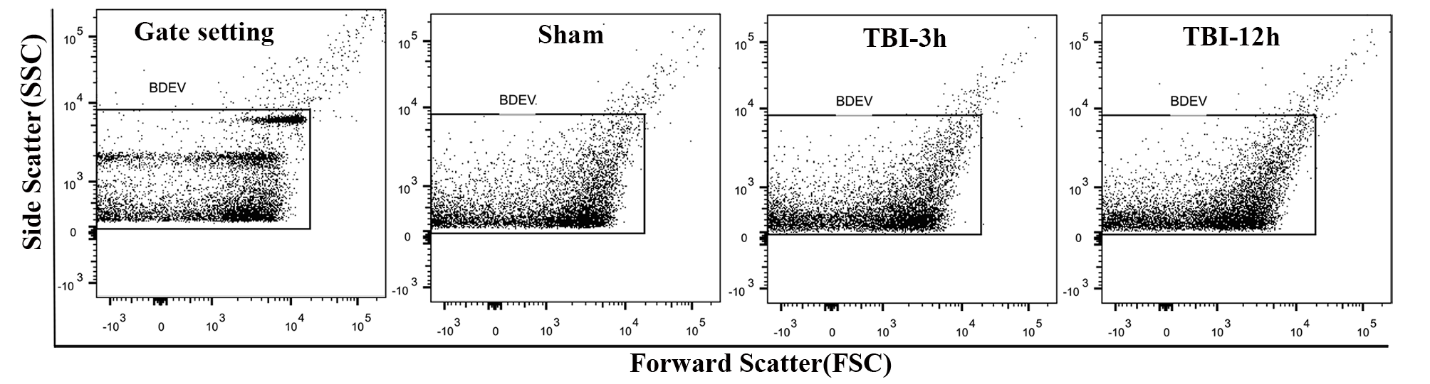

Supplement: Supplementary Materials — Supplementary Figure 1: the level of BDEV in brain markedly increased after TBI. The concentration of BDEV in brain is measured by flow cytometry in three groups (From left to right in the panel means, respectively: EV gate setting, sham group, 3 hours, and 12 hours after TBI). [file 2257427.f1.docx]
